# Supplementary material for: Somatic copy number variant load in neurons of healthy controls and Alzheimer’s disease patients
Source: Acta Neuropathol Commun. 2022 Nov 30;10:175. doi: 10.1186/s40478-022-01452-2 (PMC9714068; doi:10.1186/s40478-022-01452-2)
Supplement: Supplementary file 4 — Additional file 4: Fig. S1. Information about the samples of uncorrected and PCA-corrected data. Figure S2. CN profiles of three cells deviating from the range of 1.9 and 2 after correction. Figure S3. Examples of CNVs that failed to pass and passed the filtering criteria in the uncorrected-filtered data and PCA-corrected data. [file 40478_2022_1452_MOESM4_ESM.pdf]

Supplemental Information

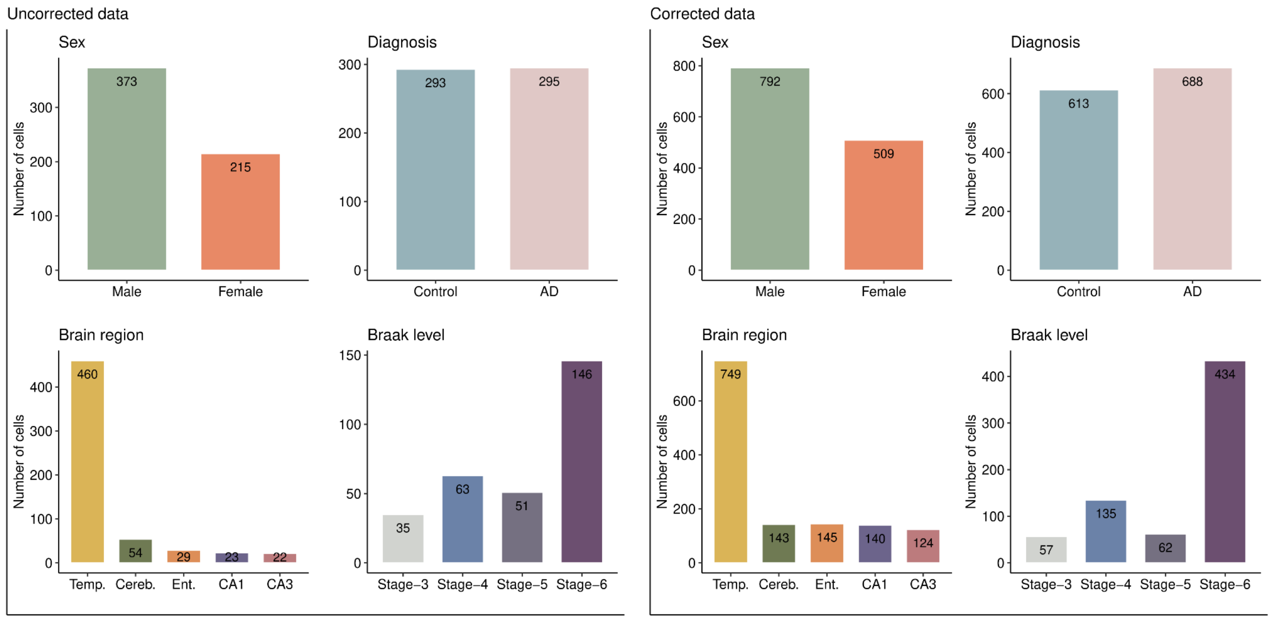

Figure S1. Information about the samples of uncorrected and PCA-corrected data. Distribution of samples according to sex (female, male) (*A*), diagnoses (Alzheimer's Disease, control) (*B*), brain regions (temporal cortex, cerebellum, entorhinal cortex, hippocampal subfields CA1, hippocampal subfields CA3) (*C*) and Braak level (stage 3, 4, 5 and 6) (*D*).

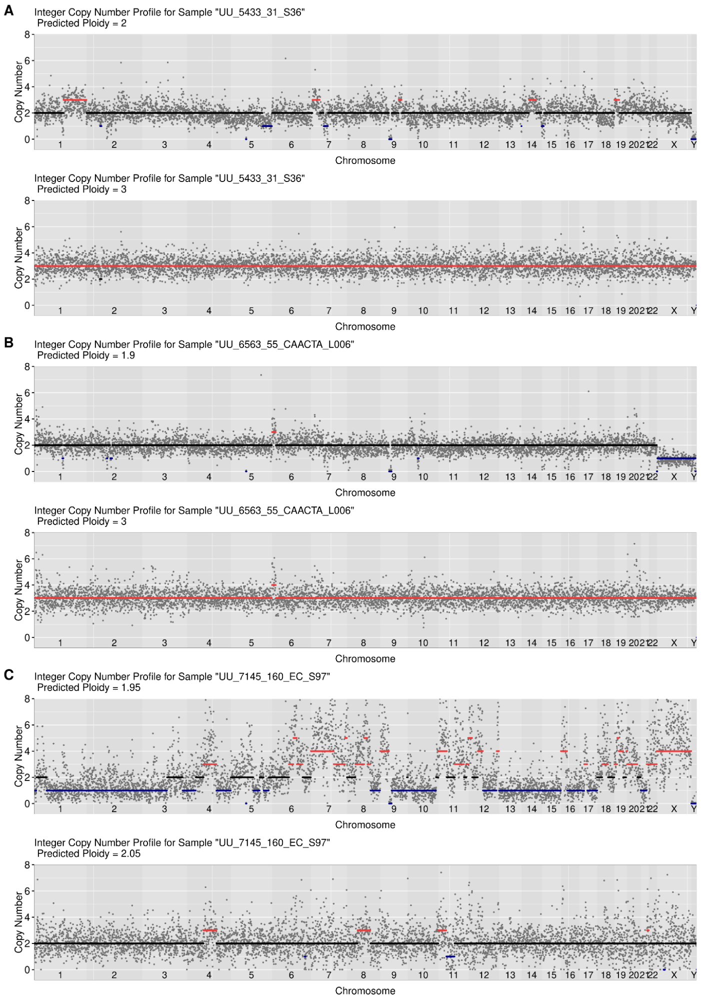

Figure S2. CN profiles of three cells deviating from the range of 1.9 and 2 after correction. The upper panels show the uncorrected data, and the lower panels show the corrected data. X-axes show chromosomes and Y-axes illustrate the CN profile of chromosomes estimated by *Ginkgo*. Each grey dot represents the scaled read counts per bin. Amplifications (CN>2) are shown in red; deletions (CN<2) in blue; disomy (CN=2) in black.

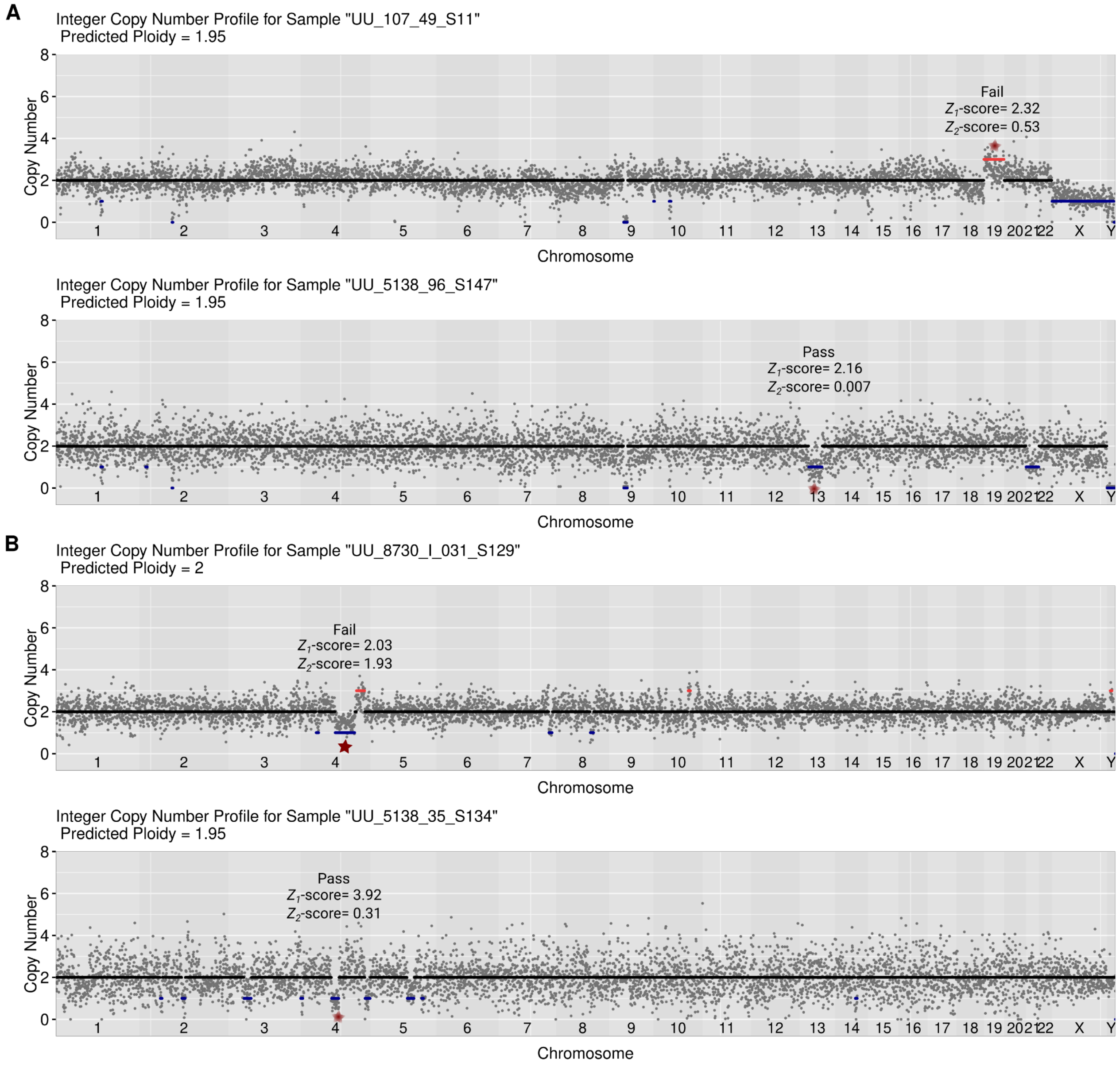

Figure S3. Examples of CNVs that (upper panels) failed to pass and (lower panels) passed the filtering criteria (Z_1-scores ≥2 and Z_2-scores ≤0.5) in the uncorrected-filtered data (*A*) and PCA-corrected data (*B*). Absolute values of the Z_1- and Z_2 scores for the CNV are indicated on the plot. The CNVs in question (that failed or passed the filtering) are marked with red stars. X-axes show chromosomes and Y-axes illustrate the CN profile of chromosomes estimated by *Ginkgo*. Each grey dot represents the scaled read counts per bin. Amplifications (CN>2) are shown in red; deletions (CN<2) in blue; disomy (CN=2) in black.
